# The association of glycemic control and fall risk in diabetic elderly: a cross-sectional study in Hong Kong

**DOI:** 10.1186/s12875-022-01807-7

**Published:** 2022-08-01

**Authors:** Long Yee Cheng, Shuk Yun Leung, Maria Kwan Wa Leung

**Affiliations:** 1 Department of Family Medicine, New Territories East Cluster, Hospital Authority, Hong Kong, China; 2 Fanling Family Medicine Centre, 1/F, Fanling Health Centre, 2 Pik Fung Road, Fanling, Hong Kong SAR, China

**Keywords:** Diabetes, Elderly, Fall, Hypoglycemia, Glycemic control

## Abstract

**Background:**

Many foreign studies investigated glycemic control and fall risk. However, there was insufficient study on this topic in Hong Kong. This study aims to find out the association of glycemic control and fall risk in the diabetic elderly in a general outpatient clinic in the North District of Hong Kong. Their frequency of falls and other associated risk factors of fall were also studied.

**Methods:**

A cross-sectional questionnaire survey was conducted on 442 diabetic patients aged 65 years-old or above with regular follow-up in a general outpatient clinic. Main outcome measure was the number of falls in the past one year from the interview date. Recurrent falls was defined as two or more falls in the past one year from the interview date. Subjects were asked about experience of hypoglycemic symptoms. HbA1c level, chronic illness, retinopathy etc. were obtained through computerized medical record review. Chi square test and logistic regression were used to assess the association between outcomes and the explanatory variables.

**Results:**

In the past one year, 23.3% participants experienced at least one fall and 8.6% had recurrent falls. Hypoglycemic symptoms, and lower visual acuity < 0.6 were significantly associated with fall (OR 2.42, *p* = 0.007 and OR 1.75, *p* = 0.038 respectively). Age 75–79 years-old had a higher likelihood of fall than the 65–69 age group (OR 2.23, *p* = 0.044). Patients with HbA1c 7.0–7.4% had a lower risk of recurrent falls when compared to those with intensive control (OR 0.32, *p *= 0.044). Other risk factors that increased risk of recurrent falls were hypoglycemic symptoms (OR 6.64, *p* < 0.001) and history of cerebral vascular accident (OR 4.24, *p* = 0.003).

**Conclusions:**

Hypoglycemic symptoms had a very strong association with falls. Less stringent HbA1c control reduced the risk of recurrent falls. Healthcare professionals need to take a more proactive approach in enquiring about hypoglycemia. There should be individualized diabetic treatment target for the diabetic elderly.

## Background

### Fall risk and consequence

Fall is a major public health concern globally. Studies conducted in Asia showed that fall rates ranged between 14.7% and 34% per annum [1–4]. The incidence of fractures after fall could be as high as 21% [4]. Other major injuries included intracranial hemorrhage, joint dislocations, and lacerations [4]. Another prospective study on Hong Kong elderly also reported significant decline in functional scores after fall [3]. Fallers had a higher probability of requiring residential care within 12 months of a fall, and a higher mortality rate [3, 5]. Apart from physical injury, there are also direct and indirect costs of fall such as health care cost, income loss and societal productivity loss [6].

### Diabetes and fall

The prevalence of fall among diabetic patients ranged from 18.8% to 78% [7–10]. Diabetic patients have higher fall risk (odds ratio [OR] 1.3 – 2.89), increased fall related hospitalization and length of stay [4, 11–17].

Factors that had been shown to increase fall risk in diabetic patients included advanced age, female, residential care, and insulin use [7, 9, 14, 18, 19]. Both intensive (Hemoglobin A1c [HbA1c] $\le$ 7.0%) or poor glycemic control (HbA1c $\ge$8.0%) had been found to have association with increased risk of fall [8, 14, 20]. Moreover, hypoglycemia and poor glycemic control were also found to increase risk of fracture and injurious fall requiring hospitalization [14, 15, 21]. On the contrary, a randomized trial found no association between intensive glycemic control and fall, and another large cohort study found that tight glycemic control (HbA1c 6.5–6.9%) was associated with lower risk of fracture in the elderly [22, 23]. Local and foreign consensus generally agreed that glycemic control should be less aggressive for the elderly, but the suggested target goal for HbA1c ranged widely, from 6.5% to 9% [24–28]. For example, a positional statement of Hong Kong suggested that the HbA1c goal can be similar to general adults in the robust elderly as long as there is no excessive hypoglycemia [28]. On the contrary, the American Geriatrics Society Guidelines recommends that the HbA1c target in older adults should be 7.5% to 8% in general. When determining the treatment goal of HbA1c of the elderly, history of hypoglycemia, other co-morbidities, functional status, and life expectancy must be considered [24–28]. Position statement of the American Diabetes Association and the European Association stated that hypoglycemia due to stringent glycemic target can lead to accidents and falls [27]. The diabetic elderly should be assessed for fall risk every 12 months or more often if needed [24].

### The local situation

When focusing on the situation in Hong Kong, one can conclude that the prevalence of both diabetes and falls are high. The 1-year prevalence of fall and recurrent falls in the Chinese elderly was about 20% and 5% respectively [1–3]. In 2014, the overall prevalence of diabetes in Hong Kong was 10.3%, and over half of them were older than 60 years old [29]. Despite these facts, there was insufficient local study on the association of HbA1c control and fall risk.

In this general outpatient clinic (GOPC) in the North District of Hong Kong, there are more than 7000 diabetic patients and half of them are over 65 years old. The Hospital Authority of Hong Kong recommends a target goal of HbA1c < 7%, and BP < 130/80 in diabetic patients [30, 31]. In this GOPC, 35% of diabetic elderly do not reach this target HbA1c. Are we going to intensify the treatment to reach the target? While physicians try to lower patients’ cardiovascular risk by targeting at a lower HbA1c level, a balance must be struck between glycemic control and fall risk. There is a need to study how hypoglycemia and HbA1c level affect falls risk in this locality. As we are facing an aging population in Hong Kong, there will be more elderlies relying on the public health service. Early identification of the high-risk group with early preventive measures will reduce their risk of fall and undoubtedly lower the burden in the public health sector.

This study aims to assess the association of glycemic control and fall risk in the diabetic elderly in a GOPC. Their frequency of falls and other probable associated risk factors of fall were studied. The null hypothesis is that hypoglycemia or HbA1c control has no association with falls.

## Method

### Study design

It is a cross sectional questionnaire survey which took place between 1^st^ November 2019 to 28^th^ February 2020.

### Setting and participants

The inclusion criteria of this study were diabetic patients aged 65 years or above, who were followed up in this GOPC, and they were ambulatory with or without walking aids. Patients who were incapable of giving informed consent were excluded. The diabetic patients who have had regular follow up in this GOPC were diagnosed by HbA1c or oral glucose tolerance test in the past. They were given an International Classification of Primary Care (ICPC) code: T90 – Diabetes non-insulin dependent. Patients aged 65 years or above with ICPC code T90 can be identified by the Clinical Data Analysis and Reporting System (CDARS) of the Hospital Authority. Random dates were picked for recruitment. All diabetic elderly who attended on those dates for follow up would be approached. Eligible patients who fulfilled the inclusion criteria were invited to attend an interview conducted by the investigator for filling informed consent and questionnaire.

### Data collection and statistical analysis

The questionnaire, designed by the author, was reviewed in the Research Committee of the Department of Family Medicine of the New Territories East Cluster. It consisted of questions on 1) number of falls in the previous one year, 2) the living arrangement, 3) use of walking aids, 4) smoking and alcohol drinking habits, and 5) history of hypoglycemic symptoms in the past one year. The questions were mainly about demographic information and health facts, so face validity was assumed. Concerning reliability, the Cronbach's Alpha score was 0.257. The principal investigator first explained to the participants the definition of fall in this study to increase the precision. A fall is defined as coming to the ground or lower level unintentionally. It is not due to an extrinsic force. If patients’ carers were present at the interview, they were asked to ascertain fall history. Patients’ medical records were reviewed to ascertain history of falls. The setup of a 1-year recall period of fall history in this study hoped to improve accuracy as 3 or 6 months were less accurate [32]. Previous studies on recall interval for falls suggested that a 1-year recall was a reliable method reaching specificity 91–95% and sensitivity 80–89% [33, 34]. Recurrent falls was defined as falling two or more times in the past one year from the interview date. Current smoker was defined as patients who have smoked in recent one month. Patients who lived alone may have a higher fall risk, thus subjects’ living arrangement was included in this study [8]. It was categorized into living alone, living with someone in the community, or living in residential home. Dependency on a walking aid was found to be a risk factor of fall in the elderly so we would also like to study on it [2, 8]. Subjects were asked whether they had experienced any hypoglycemic symptoms (feeling hungry, tiredness, dizziness, feeling trembling, palpitations) in the past one year from the interview date, regardless of whether there was blood glucose level to confirm. Computerized medical records were reviewed to ascertain history of hypoglycemic symptoms.

Each participant's medical background such as sex; age; body mass index (BMI); diabetic complication screening of retinopathy and visual acuity(VA)(defined as VA in the better-seeing eye); history of hypertension (HT), osteoarthritis (OA) of lumbar spine or lower limb joints (defined as any documentation in the past consultations notes or Xray reports), cerebral vascular accident (CVA), coronary artery disease (CAD); use of oral hypoglycemic agents (OHA) or insulin; blood test results of the latest HbA1c level within one year were collected through computerized medical record. Blood pressure (BP) was measured on the day of interview before their scheduled consultations. The number of subclasses of OHA used at the time of interview was counted. The subclasses of OHA in this study included metformin, sulphonylurea, glitazones, sodium-glucose cotransporter-2 inhibitors, dipeptidyl peptidace-4 inhibitors. The use of sulphonylurea is commonly associated with hypoglycemia in the elderly with history of fall, so it is included in the chi-square analysis [21, 35].

This study was approved by the Joint Chinese University of Hong Kong-New Territories East Cluster Clinic Research Ethics Committee (Reference number 2019.462).

### Sample size calculation

Using the CDARS of Hospital Authority, we estimated that there are 3758 diabetic patients 65 year-old or above who has regular follow up in our clinic. Based on the literature reviewed above, given a confidence level of 95% and 4.3% margin of error, the sample size needed for observing the expected proportion of falls and recurrent falls among our GOPC patients was 448 and 284 respectively. Below is the formula of sample size calculation: *n* = [z^2^* p * (1—p) / e^2^] / [1 + (z^2^ * p * (1—p) / (e^2^ * N))], where *p* = 0.43 (falls) or 0.18 (recurrent falls), z = 1.96, e = 0.043, *N* = 3758.

Data were analyzed using SPSS, version 26. Descriptive data was reported as frequencies, and percentages. Frequency distribution and normality were assessed. The data of age, BMI, HbA1c, VA were not normally distributed so they were transformed into ordinal variables. Blood pressure data was transformed into ordinal variables for easier interpretation. These data were cut so that the intervals were of equal length. As there were only 4 subjects living in the residential home, they were grouped with the “living with someone” category in the Chi square test and logistic regression. Fallers (one or more falls) were compared with non-fallers, and recurrent fallers (two or more falls) were compared with those with one or no falls.

Chi square test and logistic regression were used to assess the association between outcomes and the explanatory variables. Multivariate logistic regression was used to control for confounding factors. In the multivariate logistic regression model, all variables were included except sulphonylurea because it overlapped with OHA.

Results were expressed using the OR and 95% Confidence Intervals (95%CI). All results were considered statistically significant at *p*-value < 0.05.

## Results

### Demographic data

Four hundred forty-eight patients were invited for recruitment but 6 refused to join. A total of 442 subjects (98.7%) were recruited for the study. Table 1 shows the demographic and clinical characteristics of the participants. The number of female and male recruited was similar (223 women [50.5%]). The mean age was 74 (+—6.8) years old. Over half of the subjects were 65–74 years old. The number of subjects in the 65–69 and 70–74 age groups were similar (138 [31.2%] and 136 [30.8%] respectively). In the 75–79, 80–84 and >  = 85 age groups, there were 76 (17.2%), 49 (11.1%) and 43 (9.7%) subjects respectively. 58.6% of the participants had HbA1c < 7%. 81.9% of the subjects took OHA while only 2.9% needed insulin. 16.3% of the participants had experienced hypoglycemia symptoms. Co-morbid diseases were common, with 87.6% of the subjects having HT, 15.2% having CVA, 26.5% having OA and 9.3% having CAD. Only 4 subjects lived in the residential home. 83.9% lived with someone in the community and 15.2% lived alone. Only 22.6% of the subjects used walking aids.

**Table 1:** Demographic and clinical characteristics

								**Total Fallers** ***N* = 103**			
**Variables**	**Overall**		**Nonfaller**		**Total fallers**			**Fall once**		**Recurrent Fall**	
	***N* = 442**		***N* = 339**		***N* = 103**			***N* = 65**		***N* = 38**	
		**N%**		**N%**		**N%**			**N%**		**N%**
**Sex**											
Female	223	50.5	167	49.3	56	54.4		32	49.2	24	63.2
Male	219	49.5	172	50.7	47	45.6		33	50.8	14	36.8
**Age (years)**											
65–69	138	31.2	119	35.1	19	18.4		11	16.9	8	21.1
70–74	136	30.8	104	30.7	32	31.1		24	36.9	8	21.1
75–79	76	17.2	54	15.9	22	21.4		12	18.5	10	26.2
80–84	49	11.1	35	10.3	14	13.6		10	15.4	4	10.5
> = 85	43	9.7	27	8	16	15.5		8	12.3	8	21.1
**Current smoker**	31	7	26	7.7	5	4.9		3	4.6	2	5.3
**Drinker**	39	8.8	33	9.7	6	5.8		3	4.6	3	7.9
**Living arrangement**											
alone	67	15.2	50	14.7	17	16.5		11	17	6	15.8
lives with someone	371	83.9	288	85	83	80.6		53	81.5	30	78.9
residential home	4	0.9	1	0.3	3	2.9		1	1.5	2	5.3
**Walking aids**	100	22.6	64	18.9	36	35		24	36.9	12	31.6
**HT**	387	87.6	297	87.6	90	87.4		57	87.7	33	86.8
**CVA**	67	15.2	47	13.9	20	19.4		7	10.8	13	34.2
**OA**	117	26.5	86	25.4	31	30.1		19	29.2	12	31.6
**CAD**	41	9.3	34	10	7	6.8		4	6.2	3	7.9
**SBP (mmHg)**											
< 120	46	10.4	33	9.7	13	12.6		8	12.3	5	13.2
120–129	106	24	81	23.9	25	24.3		15	23.1	10	26.3
130–139	140	31.7	111	32.7	29	28.2		18	27.7	11	28.9
> = 140	150	33.9	114	33.6	36	34.9		24	36.9	12	31.6
**DBP (mmHg)**											
< 60	84	19	62	18.3	22	21.4		13	20	9	23.7
60–69	172	38.9	126	37.2	46	44.7		28	43	18	47.4
70–79	133	30.1	107	31.5	26	25.2		20	30.8	6	15.8
> = 80	53	12	44	13	9	8.7		4	6.2	5	13.1
**BMI**											
< 25	223	50.5	169	49.9	54	52.4		33	50.8	21	55.3
> = 25	219	49.5	170	50.1	49	47.6		32	49.2	17	44.7
**HbA1c (%)**											
< = 6.4	103	23.3	74	21.8	29	28.2		15	23	14	36.8
6.5–6.9	156	35.3	119	35.1	37	35.9		24	37	13	34.2
7.0–7.4	108	24.4	86	25.4	22	21.4		16	24.6	6	15.8
7.5–7.9	31	7	24	7.1	7	6.8		5	7.7	2	5.3
> = 8.0	44	10	36	10.6	8	7.8		5	7.7	3	7.9
**OHA**											
0	80	18.1	62	18.3	18	17.5		10	15.4	8	21.1
1	196	44.3	145	42.8	51	49.5		31	47.7	20	52.6
> = 2	166	37.6	132	38.9	34	33		24	36.9	10	26.3
**Sulfonylurea**	181	41	144	42.5	37	35.9		26	40	11	28.9
**Insulin**	13	2.9	10	2.9	3	2.9		3	4.6	0	0
**Hypoglycemia symptoms**	72	16.3	46	13.6	26	25.2		10	15.4	16	42.1
**Diabetic retinopathy**	115	26	86	25.4	29	28.2		20	30.8	9	23.7
**VA**											
< 0.6	204	46.2	141	41.6	63	61.2		39	60	24	63.2
0.6–1.0	238	53.8	198	58.4	40	38.8		26	40	14	36.8

### Number of falls

One hundred three (23.3%) participants reported falls and 38 (8.6%) participants had recurrent falls in the past one year.

### Associated risk factors for fall (one or more falls versus non-fallers)

Table 2 shows the associated risk factors for fall in the diabetic elderly. Chi-square test revealed that hypoglycemia symptoms, older age, poorer VA, and use of walking aids were significantly associated with fall. In the logistic regression analysis, only hypoglycemic symptoms, age, and poorer VA remained significantly associated with falls. Hypoglycemic symptoms were 2.4 times more likely to be associated with fall (OR 2.42, CI 1.28–4.57, *p* = 0.007). Age 75–79 years-old had a higher likelihood of fall by more than 2 times when compared to age 65–69 years old (OR 2.23, CI 1.02–4.85, *p* = 0.044). Poorer VA < 0.6 was 1.75 times more likely to cause fall (OR 1.75, CI 1.03–3.03, *p* = 0.038). There was slightly more female (54.4%) in the fallers group, but it was not statistically significant (*p* = 0.472).

**Table 2:** Risk factors for fall among diabetic elderly

Variables	Unadjusted OR	95% CI	*P* value	Adjusted OR	95% CI	*P* value
**Sex**						
Female	1.23	0.79–1.92	0.426	1.22	0.71–2.1	0.472
Male	Ref			Ref		
**Age (years)**						
65–69	Ref			Ref		
70–74	1.93	1.03–3.6	0.04*	1.86	0.96–3.61	0.067
75–79	2.55	1.28–5.1	0.008*	2.23	1.02–4.85	0.044*
80–84	2.51	1.14–5.5	0.022*	2.06	0.81–5.24	0.13
> = 85	3.71	1.69–8.14	0.001*	2.13	0.81–5.57	0.125
**Current smoker**	0.61	0.23–1.64	0.448	0.66	0.22–1.96	0.456
**Drinker**	0.57	0.23–1.41	0.305	0.69	0.26–1.84	0.454
**Living arrangement**						
alone	Ref			Ref		
with someone/residential home	0.88	0.48–1.6	0.781	0.97	0.5–1.87	0.924
**Walking aids**	2.31	1.42–3.76	0.001*	1.59	0.83–3.0	0.161
**HT**	0.98	0.50–1.9	1.000	0.88	0.41–1.85	0.729
**CVA**	1.50	0.84–2.67	0.223	1.08	0.56–2.09	0.817
**OA**	1.27	0.78–2.06	0.409	1.03	0.6–1.79	0.904
**CAD**	0.65	0.28–1.52	0.426	0.49	0.2–1.21	0.123
**SBP (mmHg)**						
< 120	Ref			Ref		
120–129	0.78	0.36–1.71	0.541	0.93	0.39–2.21	0.868
130–139	0.66	0.31–1.42	0.29	0.8	0.35–1.87	0.611
> = 140	0.8	0.38–1.69	0.56	0.87	0.36–2.08	0.749
**DBP (mmHg)**						
< 60	Ref			Ref		
60–69	1.03	0.57–1.86	0.925	1.34	0.7–2.56	0.384
70–79	0.69	0.36–1.3	0.252	1.1	0.51–2.29	0.841
> = 80	0.58	0.24–1.37	0.213	0.7	0.26–1.87	0.472
**BMI**						
< 25	Ref			Ref		
> = 25	0.90	0.58–1.4	0.73	1.02	0.62–1.67	0.95
**HbA1c (%)**						
< = 6.4	Ref			Ref		
6.5–6.9	0.79	0.45–1.4	0.423	0.82	0.45–1.52	0.535
7.0–7.4	0.65	0.35–1.23	0.188	0.55	0.28–1.11	0.094
7.5–7.9	0.74	0.29–1.92	0.54	0.71	0.25–2.04	0.525
> = 8.0	0.57	0.24–1.37	0.205	0.49	0.19–1.29	0.151
**OHA**	1.06	0.59–1.89	0.967	1.04	0.54–2.01	0.900
**Sulfonylurea**	0.76	0.48–1.2	0.284			
**Insulin**	0.99	0.27–3.66	1.000	0.63	0.15–2.65	0.526
**Hypoglycemia symptoms**	2.15	1.25–3.70	0.008*	2.42	1.28–4.57	0.007*
**Diabetic retinopathy**	1.15	0.7–1.89	0.663	1.24	0.71–2.17	0.445
**VA**						
< 0.6	2.22	1.41–3.45	0.001*	1.75	1.03–3.03	0.038*
0.6–1.0	Ref					

^*^ *p* < 0.05

Percentage of subjects with HbA1c < 7.0% was higher among fallers (64.1%) than the non-fallers (56.9%). When compared to the most intensive glycemic control (HbA1c $\le$ 6.4%), all the other HbA1c groups had lower risk of fall but none of them reached statistical significance (OR 0.49–0.82, *p* = 0.094–0.535).

Use of walking aids, chronic illnesses such as HT, CVA, OA, CAD; BP levels; BMI; retinopathy; smoking or alcohol use were not associated with increase fall risk in the logistic regression analysis.

Table 3 shows the associated risk factors for recurrent falls in the diabetic elderly. Chi-square test revealed that hypoglycemic symptoms, CVA, poorer VA were significant variables in relation to recurrent falls. Advanced age ($\ge$ 85 years-old) had a higher odd of recurrent falls when compared to age 65–69 in the chi-square test, but it lost the statistical significance in the adjusted analysis. In the logistic regression, only hypoglycemic symptoms, CVA, and HbA1c level were significant associated factors. Patients with HbA1c 7.0–7.4% had significantly lower risk of recurrent falls (OR 0.32, CI 0.1–0.97, *p* = 0.044) as compared to those with more intensive control (HbA1c $\le$ 6.4%). And this group was also appeared to have lower recurrent fall risk than the group with poor control (HbA1c $\ge$ 8.0%) (OR 0.32 vs 0.52). Those experienced hypoglycemic symptoms were 6 times more likely to have recurrent falls (OR 6.64, CI 2.69–16.4, *p* < 0.001). Patients with history of CVA had a 4-times-higher likelihood of recurrent falls (OR 4.24, CI 1.64–10.97, *p* = 0.003).

**Table 3:** Risk factors for recurrent falls among diabetic elderly. (Comparison are between subjects with No or 1 fall And those with >  = 2 falls)

Variables	Unadjusted OR	95% CI	*P* value	Adjusted OR	95% CI	*P* value
**Sex**						
Female	1.75	0.88–3.45	0.142	1.72	0.7–4.17	0.226
Male	Ref			Ref		
**Age (years)**						
65–69	Ref			Ref		
70–74	1.02	0.37–2.79	0.976	0.85	0.28–2.58	0.771
75–79	2.46	0.93–6.53	0.07	2.82	0.86–9.22	0.086
80–84	1.44	0.42–5.03	0.563	1.87	0.42–8.32	0.413
> = 85	3.71	1.3–10.6	0.014*	3.15	0.74–13.4	0.121
**Current smoker**	0.72	0.17–3.13	0.913	0.65	0.1–4.13	0.646
**Drinker**	0.88	0.26–2.99	1.0	2.12	0.46–9.69	0.332
**Living arrangement**						
alone	Ref			Ref		
with someone/residential home	0.95	0.38–2.37	1.0	1.02	0.36–2.9	0.97
**Walking aids**	1.66	0.8–3.42	0.239	0.56	0.19–1.65	0.292
**HT**	0.93	0.35–2.5	1.0	0.65	0.21–2.05	0.465
**CVA**	3.37	1.63–6.99	0.001*	4.24	1.64–10.97	0.003*
**OA**	1.31	0.64–2.7	0.579	1.47	0.62–3.53	0.383
**CAD**	0.83	0.24–2.81	1.0	0.59	0.14–2.44	0.469
**SBP (mmHg)**						
< 120	Ref			Ref		
120–129	0.85	0.28–2.66	0.785	0.88	0.23–3.38	0.853
130–139	0.7	0.23–2.13	0.529	0.92	0.25–3.42	0.906
> = 140	0.71	0.24–2.14	0.547	0.78	0.2–3.01	0.714
**DBP (mmHg)**						
< 60	Ref			Ref		
60–69	0.97	0.42–2.27	0.951	1.1	0.4–3.0	0.857
70–79	0.39	0.14–1.15	0.088	0.6	0.16–2.17	0.433
> = 80	0.87	0.27–2.75	0.81	1.08	0.26–4.45	0.919
**BMI**						
< 25	Ref			Ref		
> = 25	0.81	0.42–1.58	0.652	1.3	0.6–2.97	0.48
**HbA1c (%)**						
< = 6.4	Ref			Ref		
6.5–6.9	0.58	0.26–1.29	0.18	0.42	0.16–1.09	0.075
7.0–7.4	0.37	0.14–1.01	0.053	0.32	0.1–0.97	0.044*
7.5–7.9	0.44	0.09–2.04	0.294	0.29	0.05–1.73	0.173
> = 8.0	0.47	0.13–1.71	0.249	0.52	0.12–2.34	0.4
**OHA**	0.81	0.36–1.85	0.784	0.67	0.25–1.81	0.429
**Sulfonylurea**	0.56	0.27–1.16	0.161			
**Hypoglycemia symptoms**	4.52	2.24–9.13	< 0.001*	6.64	2.69–16.4	< 0.001*
**Diabetic retinopathy**	0.87	0.4–1.9	0.881	0.91	0.36–2.29	0.834
**VA**						
< 0.6	2.13	1.08–4.17	0.042*	1.32	0.56–3.13	0.522
0.6–1.0	Ref			Ref		

^*^p < 0.05

Use of walking aids, other chronic illnesses such as HT, OA, CAD; BP levels; BMI; retinopathy or VA; smoking or alcohol use were not associated with recurrent fall risk in the logistic regression analysis.

## Discussion

### Frequency of falls

The fall rate was alarming in this study. The result showed that 23.3% diabetic elderly reported fall in the past one year, and 8.6% had recurrent falls. The fall rate was comparable to similar studies conducted in America (23%) and Malaysia (18.8%) [7, 19]. But it was much lower than the fall rate in United Kingdom and China, which was 39% and 36% respectively [8, 12]. Both fall and recurrent falls rates were higher among the diabetic patients in this study as compared to previous study among local general elderly (23.3% vs 20% and 8.6% vs 5% respectively) [1–3, 36]. This is worth our attention to the higher percentage of fall in the diabetic patients in view of the potentially serious consequence to the individuals, care givers and society.

### Glycemic control and fall risk

This study also specifically correlated HbA1c level and fall risk. HbA1c 7.0–7.4% was significantly less likely to cause recurrent falls when compared to HbA1c $\le$6.4%. A study in America also reported that the fall risk markedly decreased when HbA1c was > 7.0% [20]. An international position statement also recommended an HbA1c target range of 7.0 -7.5% for diabetic elderly on treatment [26].

Patient with hypoglycemic symptoms had a significantly higher risk of fall and recurrent falls. The risk of fall and recurrent falls increased by 2.4-folds and 6.6-folds respectively. Similar studies also showed 2 to 4-folds increased in one-year fall risk if the diabetic elderly experienced hypoglycemia [21, 35].

In view of this, physicians should screen patients’ hypoglycemic symptoms on every visit. Appropriate measures should be imposed to minimize the chance of hypoglycemia by medication adjustment, less stringent HbA1c control in the frail elderly, education on home blood glucose monitoring and self-management of hypoglycemic attack.

It is well recognized that sulfonylurea or insulin can cause hypoglycemia [37]. They had been found to be risk factors of fall or hospitalization in other studies [11, 19, 36, 37]. However, in this study, OHA or insulin was not associated with fall. It might be due to a relatively small sample size.

### Other risk factors of fall

Age, VA and CVA were also found to be important predictors of fall in this study.

Age 75–79 years-old had higher odds of fall than age 65–69 years-old. Similar findings had been reported in the Caucasian and Southeast Asian [7, 8, 21].

VA < 0.6 increased odds of fall. Similarly, there were other studies which had shown that impaired vision increased the risk of fall [38, 39]. Diabetic retinopathy might increase fall risk, but this correlation could not be observed in this study [7, 40]. Patients with previous CVA had unsteady gait and were prone to fall [1]. This study found that they had 4-times-higher risk of recurrent falls, which is very alarming. Physicians must pay more attention to this high-risk group. Fall risk assessment should be considered in every consultation. Concerning their glucose control, a less aggressive approach should be considered for the following reasons: 1) Meta-analysis studies concluded that intensive glucose lowering e.g. targeting a lower HbA1c level below 7%, or prescribing multiple OHA, had no significant effect on risk reduction for stroke [41, 42]. 2) The American Heart Association pointed out that severe hypoglycemia could increase the risk of cardiovascular event or nonfatal stroke [43].

### Strength and limitations

The strength of this study is that the computerized record system and well-organized diabetic complication screening in GOPC provided abundant updated clinical information of the patients. The information on medications, past medical illness and HbA1c level was well recorded, which helped to reduce recall bias in these areas.

This study had several limitations. First of all, the study was conducted in one GOPC only. The medical background and diabetic control of patients were quite similar in most GOPCs and primary care clinics in Hong Kong. However, the study result might not be applicable to those complicated cases treated in the specialist clinics. Secondly, the sample size in some of the subgroups were small. Large proportion of the participants achieved HbA1c level below 7.5%, while only a few fallers had a higher HbA1c level. Therefore in the subsequent risk analysis, subgroups with higher HbA1c level could not reach a statistically significant result. The number of subjects in the insulin group was too small. Less than 3% of the participants were using insulin. Therefore, possible relationship between insulin use and fall could not be established in this study.

Thirdly, in this retrospective study, patients needed to recall their fall experience and hypoglycemic symptoms in the past one year. It was prone to recall bias which was common in many similar studies on hypoglycemia or fall [7, 23, 44]. And the temporal relationship between hypoglycemia and falls could not be established in this cross-sectional study.

Furthermore, as this study relied on a history of hypoglycemic symptoms without confirmation with blood glucose level, under- or over-estimation of hypoglycemic events was inevitable. Hypoglycemia has various symptoms, which make it difficult to be distinguished from symptoms due to other chronic illness or side effects of medications. A study found that both diabetic patients and non-diabetic patients could present with such hypoglycemia-like symptoms [45]. On the other hand, underestimation could be due to unrecognized hypoglycemia. As high as 46.6% of type 2 diabetic patients had asymptomatic hypoglycemia which was detected by continuous glucose monitoring device [46].

### Recommendations

The authors recommend further research on this topic involving both public and private clinics from different districts in Hong Kong. Further studies with larger sample size on fall among patients using different OHA groups or insulin is also recommended. To reduce recall bias and to improve the objectiveness on hypoglycemic experience, future studies may be conducted prospectively and recruit patients who have home blood glucose monitoring with glucometer.

## Conclusion

In the diabetic elderly, hypoglycemic symptoms had a very strong association with both fall or recurrent falls. Less stringent HbA1c control i.e. HbA1c 7.0–7.4% reduced the risk of recurrent falls. Aging and poor VA were associated with higher fall risk, and history of CVA were associated with more recurrent falls. Healthcare professionals need to take a more proactive approach in enquiring patients about hypoglycemic symptoms and encourage patients to have home blood glucose monitoring. There should be individualized diabetic treatment target for those advanced age patients with CVA. Patients with high fall risk should be identified early for education on fall prevention. To empower patients for better self-care, the Department of Health and Hospital Authority should promote the use of mobile apps for spot glucose monitoring.

## Data Availability

The datasets generated and/or analysed during the current study are not publicly available as the raw data is owned by the Hong Kong Hospital Authority but are available from the corresponding author on reasonable request.

## Abbreviations

HbA1c: Hemoglobin A1c
GOPC: General outpatient clinic
ICPC: International Classification of Primary Care
CDARS: Clinical Data Analysis and Reporting System
BMI: Body mass index
VA: Visual acuity
HT: Hypertension
OA: Osteoarthritis
CVA: Cerebral vascular accident
CAD: Coronary artery disease
OHA: Oral hypoglycemic agents
BP: Blood pressure
OR: Odds ratio
95%CI: 95% Confidence intervals
